# Conceptualizing Leader–Member Exchange as a Second-Order Construct

**DOI:** 10.3389/fpsyg.2022.953860

**Published:** 2022-07-07

**Authors:** Brian Manata, Siri Grubb

**Affiliations:** ^1^Department of Communication Arts and Sciences, The Pennsylvania State University, University Park, PA, United States; ^2^Department of Communication, Portland State University, Portland, OR, United States

**Keywords:** factor analysis, second-order, measurement, LMX, leader–member exchange

## Abstract

To date, scholars have focused a considerable amount of effort on developing valid measures of leader–member exchange (LMX). Although useful, it is unclear whether this proliferation in measurement is warranted. Specifically, although perhaps meaningful conceptual distinctions are made when developing new LMX measures, it is unclear whether these measures differ sufficiently from previously established measures. This manuscript explores this possibility. We begin by providing a brief review of the current state of LMX measurement, and then proceed by describing three different studies through which this research question is explored. Results suggest that virtually all measures of LMX included in this investigation are indicators of the same underlying second-order factor, i.e., they are all measuring the same construct.

## Introduction

To date, scholars have focused considerable effort on developing valid measures of leader–member exchange (LMX). In addition, these measures have been developed primarily to document the positive effects of LMX on performance and other outcomes (see Martin et al., 2016). Although useful, it is unclear whether this proliferation in measurement is warranted. Specifically, although perhaps meaningful conceptual distinctions are made when developing new LMX measures (e.g., Jian et al., 2014), it is unclear whether they differ sufficiently from measures established previously to warrant their creation. Instead, it is possible that many available LMX scales measure the same underlying latent factor, and so provide redundant information (Martin et al., 2016). This manuscript explores this possibility. We begin by providing a brief review of the current state of LMX measurement, and then describe three studies through which this general research question is explored.

### A Brief Review of Leader–Member Exchange Measurement

Identified originally by Graen et al. (1972) and Dansereau et al. (1973), LMX was first referred to as vertical dyad linkage (VDL). These scholars used this term to emphasize the inherent variance in relational quality among supervisor and subordinate dyads (e.g., Graen et al., 1972; Dansereau et al., 1973), which challenged the commonly held belief that leaders held consistent and uniform relationships with all subordinates. Over the following decade, a conceptual shift was made from VDL to LMX, emphasizing the exchange between leader and members, rather than the hierarchical relationship between supervisor and subordinate. In brief, high-quality LMX relationships are said to evidence trust, respect, and beneficial social exchanges between leaders and members, whereas low-quality LMX relationships are said to lack these important relational characteristics (see Graen and Uhl-Bien, 1995).

Early measures of this general construct included the Leader Behavior Description Questionnaire (LBDQ; Graen et al., 1972) and a varying number of items that would eventually be refined into the popular LMX-7 scale (Schriesheim et al., 1999). Subsequent advancements led to the development of alternate measures of LMX, the most popular being LMX-MDM. Introduced by Liden and Maslyn (1998), this 11-item scale measures four underlying dimensions of LMX: affect, loyalty, contribution, and professional respect. As noted by Bauer and Erdogan (2015), this scale was developed using a more rigorous process than LMX-7, the latter of which emerged and fluctuated as LMX was defined. In addition, although the distinguishing feature of the LMX-MDM scale is its multidimensionality, many researchers continue to treat this scale as unidimensional (e.g., Martin et al., 2016).

Since then, other measures of LMX have been developed to focus on aspects of the leader–member relationship that researchers consider to be absent from existing measurement approaches. For example, Leader Member Social Exchange (LMSX; Bernerth et al., 2007) emphasizes the role of social exchange; that is, when positive actions from one member of the dyad prompt feelings of indebtedness and repayment from the other member. Similarly, the economic LMX/social LMX scale (ELMX/SLMX; Kuvaas et al., 2012) emphasizes both social and economic exchanges that can occur between leaders and members. Relatedly, the leader–member conversation quality scale (LMCQ; Jian et al., 2014) measures efficiency and accuracy of information exchange between leaders and subordinates.

Although interesting conceptually, it is unclear whether extant LMX measures differ sufficiently from one another to constitute unique constructs. That is, existing measures tend to capture elements of the LMX construct as it has been conceptualized over the years, i.e., the extent to which the supervisor-subordinate relationship is of high relational and social-exchange quality (Graen and Uhl-Bien, 1995). Indeed, an inspection of LMX measures used commonly indicates that there is substantial conceptual and theoretical consonance between different scales and subscales. Liden and Maslyn (1998), for example, stipulated that affect, loyalty, contribution, and professional respect were distinct theoretical constructs, but they are essentially synonymous with LMX itself (i.e., high relational quality) (see Graen and Uhl-Bien, 1995). Similarly, the LMSX and LMCQ were intended to make up for a lack of attention to social exchange and communication, respectively, in early LMX measures, but the creation of LMX-7 was grounded on the premise that LMX represented a social-exchange process characterized by beneficial communication practices (Graen and Uhl-Bien, 1995, p. 227). The ELMX, which is perhaps the one construct that is best differentiated from traditional LMX, likewise proposed to focus specifically on the transactional nature of leader–member relationships, even though Graen and Uhl-Bien (1995) had posited that “LMX is both transactional and transformational” (p. 238). Thus, concepts that were purportedly missing from the initial LMX-7 had in many cases already been described in LMX’s theoretical framework (i.e., the same theoretical domain).

In addition to their conceptual and theoretical similarities, it is also unclear whether many of these measures are empirically distinct (see Martin et al., 2016). Concern about their distinctiveness is raised by reported correlations among different measures of LMX. For example, Liden and Maslyn (1998) reported an uncorrected correlation of *r* = 0.84 between their global measure of LMX-MDM and LMX-7. Similarly, Bernerth et al. (2007) reported that their measure of LMSX correlated strongly with both LMX-7 (*r* = 0.86) and LMX-MDM (*r* = 0.79), uncorrected for measurement error. More recently, Jian et al. (2014) reported an uncorrected correlation of *r* = 0.80 between their measure of LMCQ and LMSX. Finally, in their meta-analysis, Martin et al. (2016) showed that LMX, LMX-MDM, and other LMX measures correlated strongly (average ρ = 0.87) across myriad investigations. Evidently, many extant measures of LMX correlate strongly and positively with one another, which casts doubts on their discriminant validity (Campbell and Fiske, 1959). Instead, extant evidence raises the possibility that existing measures of LMX measure the same latent factor, suggesting they may conform to a second-order unidimensional construct.

Of the measurement work that has been reviewed thus far, only one of the investigations tested for this possibility explicitly (cf. Joseph et al., 2011). In specific, Liden and Maslyn (1998) performed a second-order factor analysis and found that all four first-order constructs in the LMX-MDM were indicators of the same higher-order latent factor. This finding, although important, was not emphasized by the authors. We suggest, however, that this finding is of decided theoretical importance, and we suggest further that it has important methodological implications. Stated differently, the possibility that extant LMX measures are second-order unidimensional is worth investigating for both empirical and theoretical reasons.

Empirically, ignoring second-order factors can lead to numerous analytical problems. First, failing to account for the existence of a second-order factor increases the probability that error terms will need to be correlated to attain adequate fit for a measurement model, which indicates that the model is either incorrect or invalid to some degree (Gerbing and Anderson, 1984). Furthermore, a causal model that ignores a second-order factor will likely fail to fit the data (Hunter and Gerbing, 1982). In either case, ignoring the presence of a second-order factor may yield unexplained residual variance, making major *post hoc* modifications more likely. Other problems that are not specific to structural equation modeling can occur because of the typically high correlations among factors that are second-order unidimensional. For example, Cohen et al. (2014) showed that regression coefficients can change in both size and direction if the included independent variables correlate too strongly with one another. Standard errors are also likely to be inflated because of multicollinearity, which may render false conclusions. Thus, if measures of LMX are in fact second-order unidimensional, especially because of the remarkably high correlations among different scales, then treating these measures as distinct will introduce a host of analytical issues. An example of ignoring a second-order factor can be found in Liden and Maslyn (1998), where the four different LMX-MDM facets were kept separate in a regression analysis despite producing evidence for a second-order factor. Perhaps unsurprisingly, the reported regression coefficients suggest some trends that are difficult to interpret. For example, professional respect—supervisor admiration—was associated negatively with supervisor performance ratings. Although there may be good theoretical reasons for such trends, they may simply be an artifact of keeping interchangeable measures separate in the analysis.

Ignoring the presence of a second-order factor is also problematic for theoretical reasons. For one, ignoring second-order factors undermines theoretical parsimony (Harter and Schmidt, 2008), which constitutes one of the cornerstones of the scientific enterprise. Second, if the measures described previously are in fact second-order unidimensional, then treating them as distinct will fail to capture the correct theoretical construct. Finally, ignoring second-order factors is problematic because it contributes to the problem of construct proliferation (Cruz and Manata, 2020). In general, construct proliferation is a problem because it can give the false impression that there are many more theoretical constructs than there actually are. Ultimately, this will create theoretical confusion and impede scientific progress. For example, Gottfredson et al. (2020) argued recently that LMX’s conceptualization and measurement has been decidedly inconsistent from scholar to scholar. Moreover, and because of this, these authors recommended abandoning the LMX construct altogether. Although such conclusions may appear warranted, one alternative interpretation is that the inconsistencies noted by Gottfredson et al. (2020) are based on the false premise that different LMX conceptualizations stem from different theoretical domains. Instead, providing evidence for a broad second-order LMX factor would mean that different conceptualizations of LMX were similar in actuality, and that the noted theoretical discrepancies were an artifact of construct proliferation.

For these reasons, the specific purpose of this investigation is to interrogate whether ostensibly different measures of LMX are measuring the same latent construct—in other words, if they are second-order unidimensional. If they are, then treating these measures as empirically distinct has likely produced erroneous or misleading results because of the analytical problems listed previously. Moreover, to the extent that LMX measures are treated as separate conceptual constructs when in fact they are not, one would expect impediments to both knowledge accumulation and the progression of science more generally (Le et al., 2010; Joseph et al., 2011).

When selecting among the available LMX measures for inclusion, a decision was made to focus on scales utilized most frequently in the LMX corpus: the LMX-7, LMX-MDM, LMSX, and ELMX/SLMX. Additionally, the LMCQ was included because it (1) was developed recently, and (2) is representative of extant LMX-based scales that are used less frequently. Information regarding these five measures is summarized in Table 1.

**TABLE 1:** Sample of extant leader–member exchange (LMX) measures and their citation counts.

Measure	Citation count	Citation
LMX-7	9044	Graen and Uhl-Bien, 1995
LMX-MDM	2931	Liden and Maslyn, 1998
LMSX	325	Bernerth et al., 2007
ELMX/SLMX	188	Kuvaas et al., 2012
LMCQ	46	Jian et al., 2014

*LMX-7, leader–member exchange; LMX-MDM, leader–member exchange-multidimensional measure; LMSX, leader–member social exchange; ELMX, economic leader–member exchange; SLMX, social leader–member exchange; LMCQ, leader–member conversation quality.*

## Study 1

### Procedure

Subjects were sampled via Qualtrics’s online sampling services, and data collection continued for approximately 1 week. All items and response scales were kept in their originally presented format.

### Sample

Organizational members were sampled from numerous organizations from various industries and professions (*N* = 315). Subjects were primarily female (*n* = 247, 78.4%), middle-aged (*M* = 35.55, *SD* = 12.14), generally white (*n* = 266, 84.4%; black: *n* = 21, 6.7%; Asian: *n* = 12, 3.8%; other/mixed: *n* = 16, 5%), and ranged in level of education (less than high school: *n* = 5, 1.6%; high school graduate or GED: *n* = 45, 14.3%; some college but no degree: *n* = 78, 24.8%; associates degree: *n* = 46, 14.6%; bachelor’s degree: *n* = 94, 29.8%; masters, doctoral, or professional degree: *n* = 47, 15%). Additionally, subjects reported working in a private-for-profit organization (*n* = 218, 69.2%), private-not-for-profit organization (e.g., charitable organization; *n* = 37, 11.7%), as well as in the local (*n* = 23, 7.3%), state (*n* = 14, 4.4%), and federal government sectors (*n* = 13, 4.1%). Finally, subjects reported working for organizations of various sizes (e.g., small = 1–4, large ≥ 1,000), a range of incomes (e.g., less than $10,000, $150,000 or more), occupying a host of positions (e.g., management, service, sales, construction, transportation, and farming), and working in numerous industries (e.g., real estate, retail trade, education services, health care or social assistant, food services, and manufacturing).

### Measures

#### LMX-7

This measure was taken from Graen and Uhl-Bien’s (1995) adaptation of Scandura and Graen’s (1984) classic measure of LMX. It is a seven-item Likert-type scale designed to be given to both leaders and subordinates to assess perceptions of LMX quality. Items are scored on a five-point scale with anchors specific to the question. For example, one item asks, “how well does your leader understand your job problems and needs,” with responses ranging from 1 (*not a bit*) to 5 (*a great deal*), whereas another item states, “I have enough confidence in my leader that I would defend and justify his/her decision if he/she were not present to do so,” with responses ranging from 1 (*strongly disagree*) to 5 (*strongly agree*).

#### Leader–Member Exchange-MDM

This measure was taken from Liden and Maslyn’s (1998) four-factor measure of LMX. The factors are affect, loyalty, contribution, and professional respect. Eleven items are scored on a scale from 1 (*strongly disagree*) to 7 (*strongly agree*). Sample items include “My supervisor is a lot of fun to work with” (affect), “My supervisor would defend me to others in the organization if I made an honest mistake” (loyalty), “I do work for my supervisor that goes beyond what is specified in my job description” (contribution), and “I am impressed with my supervisor’s knowledge of his/her job” (professional respect).

#### Leader Member Social Exchange

This measure was adopted from Bernerth et al. (2007), which contained 8 items that were scored on a scale ranging from 1 (*strongly disagree*) to 7 (*strongly agree*). Sample items include “If I do something for my manager, he or she will eventually repay me,” and “my manager and I have a two-way exchange relationship.”

#### ELMX/SLMX

These measures were taken from Kuvaas et al. (2012), which were adapted from Shore et al. (2006). Eight items were created to measure economic LMX, e.g., “my relationship with my manager is mainly based on authority, he or she has the right to make decisions on my behalf and I do what I am told to do,” and 8 items were created to measure social LMX, e.g., “my relationship with my manager is based on mutual trust.” Scores ranged from 1 (*strongly disagree*) to 5 (*strongly agree*).

#### Leader–Member Conversation Quality Scale

This measure was taken from Jian et al.’s (2014) 9-item communication-based measure of LMX. Sample items include “my supervisor and I interpret each other’s ideas accurately when discussing work-related matters,” and “when discussing work-related matters, my supervisor and I can convey a lot to each other even in a short conversation.” Scores ranged from 1 (*strongly disagree*) to 7 (*strongly agree*).

#### Additional Variables

The following outcome variables were also included in the measurement model for two primary reasons: (1) to provide additional assessments of construct parallelism; and (2) to provide evidence of criterion validity, given their relevance to the LMX construct. Of note, if the CFA provides evidence for the existence of a second-order unidimensional factor, then the effect sizes produced between each of the different LMX measures and these outcomes variables should be similar (i.e., effect size information should be relatively redundant).

##### Job Satisfaction

This measure was taken from Babin and Boles (1996), which is a shortened version of Brayfield and Rothe’s (1951) classic index of job satisfaction. The scale is designed to measure an employee’s overall attitude toward their work. Nine items are rated on a scale from 1 (*strongly disagree*) to 5 (*strongly agree*). Sample items include “Most of the time I have to force myself to go to work” and “I find real enjoyment in my work.”

##### Commitment

This 15-item measure was taken from Mowday et al. (1979) and assesses the extent to which employees feel that their goals align with those of their employer, as well as their desire to stay with the organization. Items are rated on a scale ranging from 1 (*strongly disagree*) to 7 (*strongly agree*) and sample items include “I am proud to tell others that I am part of this organization” and “I talk up this organization to my friends as a great organization to work for.”

##### Organizational Citizenship Behaviors

This measure was adapted from Smith et al. (1983) and examines prosocial behaviors that are above and beyond job requirements. Sixteen statements are rated on a scale from 1 (*strongly disagree*) to 5 (*strongly agree*). Sample items include “I help others who have heavy work loads” and “I do not spend time in idle conversation.”

### Analysis

The structural validity of the measurement model was assessed using confirmatory factor analysis (CFA) in the R software environment (Gerbing, 2016; R Core Team, 2016). Factor loadings were obtained using centroid estimation, and internal consistency and parallelism theorems were used to evaluate the construct validity of each item (Hunter and Gerbing, 1982). Specifically, internal consistency and parallelism theorems were used to generate and compare predicted versus obtained correlation coefficients, whereby large discrepancies between the two were treated as large errors and thus indicative of invalidity (Boster, 2012). Items deemed invalid by the analysis were removed before performing subsequent analyses because they were not homogeneous with the other items in their assigned factor cluster (Hunter, 1980; Anderson and Gerbing, 1988).

Model fit was further evaluated with the comparative fit index (CFI) and standardized root mean square residual (SRMR), which were calculated following the use of maximum likelihood estimation in lavaan in the R software environment (Rosseel, 2012; R Core Team, 2016). Model fit was deemed acceptable if CFI values approached 0.95 and SRMR values were at or close to 0.08 (e.g., Hu and Bentler, 1999). Moreover, if the comparison of models was necessary, the Akaike information criterion (AIC) served as an additional indicator of model fit.

### Results

#### First-Order Unidimensionality

Inspection of the initial measurement model evidenced poor fit, χ^2^(3938) = 8954.89, CFI: 0.78, SRMR: 0.09, AIC: 76913.02. In general, the fit indices produced during this analysis pointed to model misspecification. Consequently, the residual matrix was inspected with the intent of removing invalid items in the interest of improving model fit and thus construct validity (Hunter and Gerbing, 1982; Anderson and Gerbing, 1988; Boster, 2012).

Inspection of the residual matrix indicated the existence of numerous problematic items across each of the factors (i.e., items that lacked validity). Thus, these items were removed from the measurement model, and an additional CFA was performed to assess the fit of this abridged model (for a list of retained items, see Table 2).

**TABLE 2:** Subordinate items and factor loadings.

	Factor loadings	
	
	Study 1	Study 2
*LMX-7*	*0.76*	*0.73*
Do you know where you stand with your leader. Do you usually know how satisfied your leader is with what you do?	0.65	0.61
Again, regardless of the amount of formal authority your leader has, what are the chances that he/she would “bail you out,” at his/her expense?	0.74	0.71
How would you characterize your working relationship with your leader?	0.70	0.70
*LMX-MDM*		
*Affect*	*0.84*	*0.83*
I like my supervisor very much as a person.	0.92	0.77
My supervisor is the kind of person one would like to have as a friend.	0.92	0.77
*Loyalty*	*0.87*	*0.82*
My supervisor would come to my defense if I were “attacked” by others	0.89	0.80
My supervisor would defend me to others in the organization if I made an honest mistake	0.89	0.80
*Contribution*	*0.67*	*0.75*
I do work for my supervisor that goes beyond what is specified in my job description	0.80	0.65
I am willing to apply extra efforts, beyond those normally required, to further the interests of my work group	0.80	0.65
*Professional respect*	*0.80*	*0.83*
I am impressed with my supervisor’s knowledge of his/her job	0.91	0.89
I respect my supervisor’s knowledge of and competence on the job	0.95	0.85
I admire my supervisor’s professional skills	0.87	0.88
*LMSX*	*0.88*	*0.85*
I do not have to specify the exact conditions to know my manager will return a favor	0.84	0.75
I have a balance of inputs and outputs with my manager	0.79	0.85
My efforts are reciprocated by my manager	0.91	0.86
*ELMX*	–	–
I do what my manager demands from me, mainly because he or she is my formal boss	0.74	0.68
I do not care what my manager does for me in the long run, only what he or she does right now	0.67	0.47
My relationship with my manager is mainly based on authority, he or she has the right to make decisions on my behalf and I do what I am told to do	0.83	0.69
All I really expect from my manager is that he or she fulfills his or hers formal role as supervisor or boss	0.77	0.67
*SLMX*	*0.70*	*0.69*
I don’t mind working hard today–I know I will eventually be rewarded by my manager	0.74	0.56
My relationship with my manager is about mutual sacrifice, sometimes I give more than I receive and sometimes I receive more than I give	0.64	0.64
Even though I may not always receive the recognition from my manager I deserve, I know that he or she will take good care of me in the future	0.85	0.78
*LMCQ*	*0.82*	*0.83*
When talking about work tasks, the conversations between my supervisor and me are often smooth	0.78	0.74
When talking about how to get things done at work, my supervisor and I usually align our ideas pretty easily	0.91	0.80
When we discuss how to get things done at work, my supervisor and I usually have no problem correctly understanding each other’s ideas	0.92	0.80
My supervisor and I interpret each other’s ideas accurately when discussing work-related matters	0.86	0.77

*For a list of factor abbreviations, see Table 1. Factor loadings in italics represent the trait factor loadings from the second-order analysis. The complete measures can be found in Supplementary Table 1.*

Upon removal of these invalid items, model fit improved, χ^2^(563) = 880.70, CFI: 0.96, SRMR: 0.04, AIC: 32652.69. Specifically, the CFI and SRMR met their stipulated cutoff values, and the AIC also evidenced a notable improvement. In addition, model fit remains adequate when the fit of the LMX measures is evaluated independent of the outcome variables, χ^2^(263) = 420.76, CFI: 0.98, SRMR: 0.03, AIC: 21547.69; this indicates that the inclusion of the auxiliary variables was not inflating the fit of the model. Finally, inspection of a model in which all retained items are made to load on a single factor provides a very poor fit to the data, χ^2^(629) = 3534.95, CFI: 0.66, SRMR: 0.10, AIC: 33725.67. This indicates that treating each of the first-order constructs as distinct provides a better fit to the data when compared to a one-factor model.

In addition to examining the extent to which the items evidence both internal consistency and parallelism, reliability for each of the factors was also investigated using coefficient α. Each of the measures’ respective α’s were all deemed acceptable by conventional standards (α: 0.72–0.94; Nunnally et al., 1967).

In sum, extant evidence indicates that the abridged model is superior psychometrically to the measurement model proposed initially; consequently, the abridged model was preferred to the measurement model proposed originally. All items retained in the analysis can be found in Table 2, and the complete measures can be found in Supplementary Table 1. Additionally, correlation coefficients, reliability coefficients, and descriptive statistics can be found in Table 3.

**TABLE 3:** Correlations, alphas, means, and standard deviations (Study 1).

	1	2	3	4	5	6	7	8	9	10	11	12	*M*	*SD*
LMX-7	(0.80)												3.60	0.91
MDM_A_	0.67	(0.91)											5.43	1.46
MDM_L_	0.68	0.74	(0.89)										5.49	1.32
MDM_C_	0.50	0.56	0.64	(0.78)									5.58	1.20
MDM_PR_	0.57	0.72	0.66	0.51	(0.94)								5.52	1.44
LMSX	0.68	0.71	0.75	0.56	0.69	(0.88)							4.99	1.34
ELMX	−0.19	−0.20	−0.18	−0.16	−0.11	−0.14	(0.84)						3.09	1.03
SLMX	0.53	0.53	0.57	0.41	0.59	0.67	0.05	(0.78)					3.45	0.93
LMCQ	0.59	0.72	0.70	0.50	0.68	0.70	−0.08	0.57	(0.92)				5.55	1.23
OCB	0.32	0.25	0.39	0.53	0.23	0.32	−0.09	0.28	0.32	(0.72)			3.86	0.81
JS	0.32	0.36	0.35	0.31	0.35	0.39	−0.38	0.26	0.36	0.20	(0.89)		3.61	1.12
COMM	0.52	0.56	0.60	0.50	0.53	0.67	−0.15	0.52	0.58	0.37	0.57	(0.87)	5.13	1.34

*Reliability coefficients have been inserted in the diagonals. Correlations are not corrected for measurement error. Listwise N = 314. OCB, organizational citizenship behaviors; JS, job satisfaction; COMM, commitment. For other factor abbreviations, see Table 1.*

#### Second-Order Unidimensionality

To assess whether the 9 different LMX measures fit a second-order unidimensional factor (i.e., the extent to which they were all driven by the same underlying latent factor), the 9 LMX measures were combined into one factor cluster. Moreover, the additional outcome variables were also included in the measurement model as first-order unidimensional factors for the purposes of assessing construct parallelism (Hunter and Gerbing, 1982).

Inspection of this model suggested adequate fit, χ^2^(164) = 436.36, CFI: 0.93, SRMR: 0.06, AIC: 17164.75, but inspection of the residual matrix indicated that the ELMX factor was contributing substantial error consistently to the model. This observation is also corroborated by the fact that the ELMX factor correlated negatively and consistently with the other 8 LMX factors (see Table 3). Moreover, the factor loading for the ELMX factor was negative, thus further supporting the claim that the model was specified incorrectly. Consequently, the ELMX factor was removed from the second-order cluster, and a subsequent CFA was performed on the abridged model. That is, the ELMX item was removed from the LMX second-order cluster because its content was not homogeneous with the content of the other first-order LMX factors (Anderson and Gerbing, 1988).

Removal of the ELMX factor improved model fit, χ^2^(146) = 354.59, CFI: 0.95, SRMR: 0.05, AIC: 16262.66. Moreover, model fit remains adequate when the fit of the second-order factor is analyzed independent of the outcome variables, χ^2^(20) = 61.93, CFI: 0.98, SRMR: 0.03, AIC: 6422.01. In contrast, a model in which all retained items are made to load on one factor provided a very poor fit to the data, χ^2^(152) = 1205.27, CFI: 0.72, SRMR: 0.11, AIC: 17101.34. Consequently, extant evidence suggests that upon the removal of the ELMX factor, the other 8 LMX factors are all indicators of the same underlying latent factor, i.e., all 8 LMX measures are measuring the same construct and are thus interchangeable (Hunter and Gerbing, 1982). Moreover, the four-factor model provided a better fit to the data when compared to the one-factor model. Correlation coefficients, reliability coefficients, and descriptive statistics for each of the factors can be found in Table 4.

**TABLE 4:** Correlations, alphas, means, and standard deviations (Study 1).

	1	2	3	4	5	*M*	*SD*
LMX	(0.93)					3.60	0.91
OCB	0.40	(0.72)				3.86	0.81
JS	0.41	0.20	(0.89)			3.61	1.12
COMM	0.68	0.37	0.57	(0.87)		5.14	1.34
ELMX	−0.16	−0.09	−0.38	−0.15	(0.84)	3.09	1.03

*Reliability coefficients have been inserted in the diagonals. Correlations are not corrected for measurement error. Listwise N = 314. LMX is a composite of all other LMX variables not listed herein. For factor abbreviations, see Tables 1, 3.*

### Brief Discussion

The analyses reported herein present a synthesis of the LMX literature and construct. Specifically, although first-order unidimensionality was established for each of the factors, a second-order model also provided a good representation of the data. That is, these analyses suggest that upon removal of the ELMX factor, each of the eight remaining LMX measures can be classified as different facets of the same underlying factor; they are all measuring the same thing. Importantly, this provides some evidence for the contention that many of the measures developed since the introduction of LMX-7 are drawn from the same theoretical content domain. In addition, these results contradict the contention that transformational and transactional leadership form a part of the same construct, which align strongly with the conclusions drawn by Kuvaas et al. (2012).

Admittedly, adequate model fit was only attainable upon dropping numerous items from the measurement model. Although we do not regard the practice of dropping items as a serious limitation, it is unclear whether the preferred factor structures established in Study 1 would replicate if assessed a second time using an alternate sample. Stated differently, the resultant factor structure may have been a result of sampling error (Anderson and Gerbing, 1988). Consequently, an additional study was conducted with the intent of replicating the results produced in Study 1. Specifically, for Study 2, we predicted that dropping the same items would produce a better fitting model than the factor structure proposed originally. We also predicted that the ELMX factor would fail to fit in the second-order model.

## Study 2

Aside from the sample, the procedure, measures, and planned analyses remained identical to those used and established for study 1. The additional sample is described below, and the results of the CFAs are reported shortly thereafter.

### Sample

Like study 1, subjects were sampled via Qualtrics online sampling services (*N* = 304). Subjects were generally female (*n* = 193, 65.5%), young adults (*M* = 23.81, *SD* = 4.49), generally white (*n* = 171, 56.3%; black: *n* = 57, 18.8%; Asian: *n* = 37, 12.2%; other/mixed: *n* = 39, 12.8%), and ranged in level of education (less than high school: *n* = 8, 2.6%; high school graduate or GED: *n* = 43, 14.1%; some college but no degree: *n* = 95, 31.3%; associates degree: *n* = 54, 17.8%; bachelor’s degree: *n* = 78, 25.7%; masters, doctoral, or professional degree: *n* = 26, 8.6%). Additionally, subjects reported working in a private-for-profit organization (*n* = 218, 69.2%), private-not-for-profit organization (e.g., charitable organization; *n* = 37, 11.7%), as well as in the local (*n* = 23, 7.3%), state (*n* = 14, 4.4%), and federal government sectors (*n* = 13, 4.1%). Finally, subjects reported working for organizations of various sizes (e.g., small = 1–4, large ≥ 1,000), a range of incomes (e.g., less than $10,000, $150,000 or more), occupying a host of positions (e.g., management, service, sales, construction, transportation, and farming), and working in numerous industries (e.g., real estate, retail trade, education services, health care or social assistant, food services, and manufacturing).

### Results

#### First-Order Unidimensionality

Inspection of the initial measurement model evidenced poor fit, χ^2^(3938) = 8286.32, CFI: 0.75, SRMR: 0.09, AIC: 81585.46. Of note, a direct comparison between both sets of fit indices produced in studies 1 and 2 indicates that they are rather comparable. In testing our prediction that model fit could be improved upon by removing the same items that were removed in study 1, the same items that were removed in study 1 were removed here, and an additional CFA was performed to assess model fit.

As predicted, and comparable to study 1, model fit improved noticeably upon removal of the same items that were dropped in study 1, χ^2^(563) = 956.02, CFI: 0.94, SRMR: 0.05, AIC: 33969.32. Moreover, when the fit of the LMX measures is evaluated independent of the outcome variables, model fit remains adequate, χ^2^(263) = 444.92, CFI: 0.96, SRMR: 0.05, AIC: 23063.06. Finally, inspection of a model in which all retained items are made to load on a single factor provides a poor fit to the data, χ^2^(629) = 2123.82, CFI: 0.75, SRMR: 0.08, AIC: 34089.86; this indicates that treating each of the first-order constructs as distinct provides a better fit to the data when compared to a one-factor model.

Interestingly, despite replicating the extent to which the measurement model fit the data, the same could not be said about the reliability coefficients produced using this sample. Specifically, although most of the factors evidenced acceptable levels of reliability (see Table 5), the third LMX-MDM factor (i.e., contribution) evidenced lower reliability than is desired typically (α = 0.59). Although this type of measurement error can be corrected for (Nunnally et al., 1967), this speaks to the general dangers of opting for and implementing 2-item measures. Although, as it will be shown, the severity of this problem is mitigated by treating LMX as a second-order unidimensional factor, in part because the second-order factor is comprised of 8 factors (i.e., 8 items). Correlation coefficients, reliability coefficients, and descriptive statistics can be found in Table 5.

**TABLE 5:** Correlations, alphas, means, and standard deviations (Study 2).

	1	2	3	4	5	6	7	8	9	10	11	12	*M*	*SD*
LMX-7	(0.71)												3.55	0.86
MDM_A_	0.66	(0.75)											4.34	1.13
MDM_L_	0.56	0.66	(0.78)										4.89	1.46
MDM_C_	0.54	0.62	0.69	(0.59)									4.97	1.35
MDM_PR_	0.58	0.68	0.71	0.70	(0.81)								5.19	1.29
LMSX	0.64	0.69	0.74	0.61	0.68	(0.85)							4.85	1.36
ELMX	0.05	0.04	0.10	0.07	0.11	0.13	(0.72)						3.48	0.82
SLMX	0.51	0.57	0.54	0.45	0.53	0.63	0.31	(0.70)					3.49	0.89
LMCQ	0.61	0.68	0.64	0.56	0.68	0.73	0.14	0.61	(0.86)				5.06	1.33
OCB	0.37	0.43	0.31	0.39	0.45	0.39	0.16	0.36	0.45	(0.73)			3.66	0.85
JS	0.38	0.43	0.40	0.33	0.42	0.33	−0.20	0.23	0.40	0.21	(0.79)		3.34	1.05
COMM	0.51	0.62	0.61	0.49	0.62	0.60	0.12	0.58	0.64	0.45	0.46	(0.89)	4.85	1.42

*Reliability coefficients have been inserted in the diagonals. Correlations are not corrected for measurement error. Listwise N = 304. For factor abbreviations, see Tables 1, 3.*

#### Second-Order Unidimensionality

Analysis of the second-order measurement model proceeded identically to the analysis and procedure described in study 1. Inspection of the second-order model suggested adequate fit, χ^2^(164) = 380.38, CFI: 0.94, SRMR: 0.05, AIC: 16901.36. Nevertheless, inspection of the residual matrix indicated once again that the ELMX factor was contributing substantial error consistently to the model. Moreover, the factor loading was small (0.15), thus indicating that it was a decidedly weak indicator of the second order LMX factor. Consequently, and like study 1, this factor was removed from the analysis because the content of this factor was not homogeneous with the content of the other first-order LMX factors.

As predicted, removal of the ELMX factor from the measurement model improved model fit, χ^2^(146) = 313.73, CFI: 0.95, SRMR: 0.04, AIC: 16161.98. Moreover, when the fit of the second-order factor model is analyzed independent of the additional outcome variables, model fit remains adequate, χ^2^(20) = 75.22, CFI: 0.97, SRMR: 0.03, AIC: 6125.42. Finally, a model in which all retained items are made to load on one factor provided a poor fit to the data, χ^2^(152) = 849.56, CFI: 0.80, SRMR: 0.08, AIC: 16685.81, thus indicating that the four-factor model provided a better fit to the data when compared to the one-factor model. Consequently, the results of study 1 were replicated, and the conclusions established previously were corroborated. Correlation coefficients, reliability coefficients, and descriptive statistics can be found in Table 6.

**TABLE 6:** Correlations, alphas, means, and standard deviations.

	1	2	3	4	5	*M*	*SD*
LMX	(0.93)					3.60	0.91
OCB	0.48	(0.73)				3.66	0.85
JS	0.45	0.21	(0.79)			3.34	1.05
COMM	0.71	0.45	0.46	(0.89)		4.85	1.42
ELMX	0.14	0.16	−0.20	0.12	(0.72)	3.48	0.82

*Reliability coefficients have been inserted in the diagonals. Correlations are not corrected for measurement error. Listwise N = 304. LMX is a composite of all other LMX variables not listed herein. For factor abbreviations, see Tables 1, 3.*

### Brief Discussion

As in Study 1, Study 2 synthesizes the LMX corpus by offering a second-order conceptualization of the LMX construct. Specifically, the study replicated the findings of Study 1, reaffirming the general notions that (1) LMX scholars have been producing different measures of the same construct and (2) transformational and transactional aspects of the supervisor-subordinate relationship constitute unique constructs.

This is not to say, however, that additional measurement work is no longer useful; in fact, the opposite is true. For example, only subordinate perceptions of the leader–member relationship were solicited in Study 1 and 2. It is unclear whether the factor structure presented herein would replicate when soliciting the responses of supervisors. This is especially the case given that item content must be modified to measure supervisor perceptions of subordinate behavior. Given that LMX is a dyadic-level phenomenon (Graen and Uhl-Bien, 1995), it would be useful to understand both perspectives, i.e., it would be useful to know whether the resultant factor structure remains similar when the item referent is altered. This issue is also important to consider because previous research has demonstrated a lack of convergence between subordinate and supervisor reports of LMX relationships (Zhou and Schriesheim, 2009).

To address this issue and extend the utility and generalizability of the measurement model presented herein, a third study was conducted in which item content was altered to focus on supervisor perceptions of their subordinate exchange relationships. To transform the item content, we used Greguras and Ford’s (2006) parallel approach. To date, supervisor perceptions of LMX have been measured by adapting existing scales using either mirror or parallel approaches (Liden et al., 2016). Whereas mirrored scales aim to corroborate subordinate perceptions, parallel scales assess the dyadic relationship from the supervisor’s perspective by making “minor adaptations intended to transform items from the subordinate’s perspective to the supervisor’s perspective” (Greguras and Ford, 2006, p. 446). Given that the parallel approach was designed specifically to capture the dyadic nature of LMX from supervisor and subordinate perspectives alike, it was adopted herein.

## Study 3

Aside from the sample, which focused exclusively on supervisors, the procedure, constructs, and planned analyses remained identical to those used and established for Study 1 and 2. Moreover, and as described previously, the parallel approach was used when altering item content. To ensure that all subjects were employed in a supervisory role, all subjects were asked to indicate whether they were in a role in which they supervised others. Of note, only subjects that responded yes to this item were kept in the sample for further analysis. Moreover, when answering the survey questions regarding leader–member content, subjects were instructed to think of the subordinate that they relied on the most.

### Sample

Like studies 1 and 2, subjects were sampled via Qualtrics online sampling services (*N* = 315). Subjects were generally female (*n* = 199, 63.2%), middle-aged (*M* = 39.63, *SD* = 11.37), generally white (*n* = 242, 76.8%; black: *n* = 33, 10.5%; Asian: *n* = 17, 5.4%; other/mixed: *n* = 23, 7.3%), and ranged in level of education (less than high school: *n* = 2, 0.6%; high school graduate or GED: *n* = 39, 12.4%; some college but no degree: *n* = 54, 17.1%; associates degree: *n* = 47, 14.9%; bachelor’s degree: *n* = 99, 31.4%; masters, doctoral, or professional degree: *n* = 74, 23.5%). Additionally, subjects reported working in a private-for-profit organization (*n* = 221, 70.4%), private-not-for-profit organization (e.g., charitable organization; *n* = 35, 11.1%), as well as in the local (*n* = 22, 7%), state (*n* = 9, 2.9%), and federal government sectors (*n* = 15, 4.8%). Finally, subjects reported working for organizations of various sizes (e.g., small = 1–4, large ≥ 1,000), a range of incomes (e.g., less than $10,000, $150,000 or more), occupying a host of positions (e.g., management, service, sales, construction, transportation, and farming), and working in numerous industries (e.g., real estate, retail trade, education services, health care or social assistant, food services, and manufacturing).

### Results

#### First-Order Unidimensionality

Inspection of the initial model indicated poor fit, χ^2^(3938) = 9584.51, CFI: 0.74, SRMR: 0.13, AIC: 78328.58. Moreover, the decided lack of fit reported in this analysis is comparable to the lack of fit reported in Studies 1 and 2. As such, the same items removed in Studies 1 and 2 were removed.

Model fit improved upon removal of these items, χ^2^(563) = 908.74, CFI: 0.96, SRMR: 0.05, AIC: 31627.49. Moreover, when the fit of the LMX measures is evaluated independent of the outcome variables, model fit remains adequate, χ^2^(263) = 519.55, CFI: 0.95, SRMR: 0.05, AIC: 22170.16. Finally, inspection of a model in which all retained items are made to load on a single factor provides a very poor fit to the data, χ^2^(629) = 3746.01, CFI: 0.59, SRMR: 0.12, AIC: 34332.63.

The reliability of the measures was also investigated using coefficient α. Each of the measures’ respective α’s were all deemed adequate by conventional standards (α: 0.69–0.93). Of note, the third LMX-MDM factor (i.e., contribution) faired decidedly better when compared to the reliability coefficient reported in study 2 (α = 0.80 versus α = 0.59, respectively). Consequently, and like Studies 1 and 2, the abridged model was preferred to the originally proposed measurement model. Factor loadings and item content can be found in Table 7; complete measures can be found in Supplementary Table 1. Moreover, correlation coefficients, reliability coefficients, and descriptive statistics can be found in Table 8.

**TABLE 7:** Supervisor items and factor loadings.

	Factor loadings
	
	Study 3
*LMX-7*	*0.71*
Do you know where you stand with your subordinate. Do you usually know how satisfied your subordinate is with what you do?	0.49
Again, regardless of the amount of formal authority your subordinate has, what are the chances that he/she would “bail you out,” at his/her expense?	0.56
How would you characterize your working relationship with your subordinate?	0.97
*LMX-MDM*	
*Affect*	*0.87*
I like my subordinate very much as a person.	0.87
My subordinate is the kind of person one would like to have as a friend.	0.87
*Loyalty*	*0.84*
My subordinate would come to my defense if I were “attacked” by others	0.88
My subordinate would defend me to others in the organization if I made an honest mistake	0.88
*Contribution*	*0.72*
I do work for my subordinate that goes beyond what is specified in my job description	0.81
I am willing to apply extra efforts, beyond those normally required, to further the interests of my work group	0.81
*Professional respect*	*0.85*
I am impressed with my subordinate’s knowledge of his/her job	0.87
I respect my subordinate’s knowledge of and competence on the job	0.94
I admire my subordinate’s professional skills	0.89
*LMSX*	*0.87*
I do not have to specify the exact conditions to know my subordinate will return a favor	0.76
I have a balance of inputs and outputs with my subordinate	0.86
My efforts are reciprocated by my subordinate	0.88
*ELMX*	–
I support my subordinate, mainly because that is my job	0.79
I do not care what my subordinate does for me in the long run, only what he/she does right now	0.80
My relationship with my subordinate is mainly based on authority, I have the right to make decisions on his/her behalf	0.73
All I really expect from my subordinate is that he/she fulfills his/her formal role	0.63
*SLMX*	*0.53*
I don’t mind working hard today–I know I will eventually be rewarded by my subordinate	0.61
My relationship with my subordinate is about mutual sacrifice, sometimes I give more than I receive and sometimes I receive more than I give	0.70
Even though I may not always receive the recognition from my subordinate I deserve, I know that he or she will take good care of me in the future	0.75
*LMCQ*	*0.73*
When talking about work tasks, the conversations between my subordinate and me are often smooth	0.77
When talking about how to get things done at work, my subordinate and I usually align our ideas pretty easily	0.86
When we discuss how to get things done at work, my subordinate and I usually have no problem correctly understanding each other’s ideas	0.82
My subordinate and I interpret each other’s ideas accurately when discussing work-related matters	0.77

*For a list of factor abbreviations, see Table 1. Factor loadings in italics represent the trait factor loadings from the second-order analysis. The complete measures can be found in Supplementary Table 1.*

**TABLE 8:** Correlations, alphas, means, and standard deviations (Study 3).

	1	2	3	4	5	6	7	8	9	10	11	12	*M*	*SD*
LMX-7	(0.69)												4.14	0.81
MDM_A_	0.63	(0.86)											5.65	1.26
MDM_L_	0.62	0.76	(0.87)										5.58	1.30
MDM_C_	0.50	0.60	0.65	(0.80)									5.70	1.13
MDM_PR_	0.57	0.76	0.71	0.63	(0.93)								5.71	1.22
LMSX	0.61	0.74	0.71	0.60	0.76	(0.87)							5.41	1.21
ELMX	0.08	−0.01	−0.01	0.05	0.02	0.10	(0.83)						3.14	1.07
SLMX	0.33	0.42	0.41	0.32	0.40	0.54	0.48	(0.73)					3.60	0.86
LMCQ	0.52	0.60	0.61	0.49	0.60	0.66	0.03	0.49	(0.88)				5.76	1.11
OCB	0.37	0.37	0.38	0.46	0.33	0.38	0.10	0.27	0.36	(0.71)			4.00	0.76
JS	0.03	0.12	0.14	0.06	0.16	0.01	−0.40	−0.17	0.11	−0.05	(0.89)		3.57	1.23
COMM	0.41	0.48	0.43	0.37	0.51	0.50	0.13	0.37	0.51	0.30	0.31	(0.91)	5.40	1.40

*Reliability coefficients have been inserted in the diagonals. Correlations are not corrected for measurement error. Listwise N = 315. OCB, organizational citizenship behaviors; JS, job satisfaction; COMM, commitment. For other factor abbreviations, see Table 1.*

#### Second-Order Unidimensionality

Inspection of the second-order model suggested less-than-ideal fit, χ^2^(164) = 513.11, CFI: 0.91, SRMR: 0.08, AIC: 17008.22. As such, the residual matrix was inspected in order to determine if specific items were contributing error consistently to the measurement model. Moreover, because the ELMX factor attenuated model fit in the previous two analyses, it was expected that this factor would also cause problems here.

In replicating the results reported previously, inspection of the residual matrix indicated that the ELMX factor was contributing substantial error consistently to the model. Moreover, the factor loading was small (0.07), thus indicating that it was a decidedly weak indicator of the second order LMX factor. Consequently, this factor was removed from the analysis and a subsequent CFA was performed.

Removal of the ELMX factor improved model fit, χ^2^(146) = 324.29, CFI: 0.98, SRMR: 0.05, AIC: 16070.20. Moreover, when the fit of the second-order factor model is analyzed independent of the additional outcome variables, model fit remains adequate, χ^2^(20) = 56.78, CFI: 0.98, SRMR: 0.03, AIC: 6040.39. Finally, a model in which all retained items are made to load on one factor provided a very poor fit to the data, χ^2^(152) = 1601.61, CFI: 0.61, SRMR: 0.14, AIC: 17335.51. In consequence, the second-order model produced in Studies 1 and 2 was replicated in this study, and the conclusions established previously were corroborated once again. Correlation coefficients, reliability coefficients, and descriptive statistics for each of the factors can be found in Table 9.

**TABLE 9:** Correlations, alphas, means, and standard deviations (Study 3).

	1	2	3	4	5	*M*	*SD*
LMX	(0.92)					5.19	0.90
OCB	0.46	(0.71)				4.00	0.76
JS	0.08	−0.05	(0.89)			3.57	1.23
COMM	0.56	0.30	0.31	(0.91)		5.38	1.40
ELMX	0.10	0.10	−0.40	0.13	(0.83)	3.14	1.07

*Reliability coefficients have been inserted in the diagonals. Correlations are not corrected for measurement error. Listwise N = 315. LMX is a composite of all other LMX variables not listed herein. For factor abbreviations, see Tables 1, 3.*

## General Discussion

The second-order measurement model presented in these three studies offers a synthesis of the LMX construct. Moreover, this synthesis extends to situations in which either subordinates or supervisors are surveyed about their exchange relationships. Aside from ELMX, the results suggest that any of the eight LMX measures may be used to measure LMX (i.e., effect size information will generally be similar). In addition, these conclusions remain the same for subordinate and supervisor samples alike.

The conclusions drawn herein are generally consonant with the conclusions drawn by other scholars in this corpus (e.g., Joseph et al., 2011; Martin et al., 2016). Our work departs from others, however, in that it (a) stipulates additional LMX traits that conform to a second-order unidimensional LMX construct and (b) provides explicit tests of dimensionality for both first- and second-order portions of the construct. As such, we believe that this work makes a number of notable contributions to the LMX corpus, the largest of which is theoretical. In specific, the second-order measurement model simultaneously simplifies the LMX corpus and establishes firmly that LMX is a construct with considerable breadth. That is, LMX can be conceptualized as the degree to which the supervisor-subordinate relationship is of high relational and social-exchange quality. Moreover, these results provide strong evidence against the notion that LMX is similar to transactional forms of leadership. Instead, this work indicates that employees are able to distinguish between social and economic forms of leadership (e.g., ELMX), which supports the conclusions and theoretical arguments of Kuvaas et al. (2012) (see also Shore et al., 2006). Future scholarship is encouraged to continue considering the merits of conceptualizing LMX and leadership in this parsimonious manner (i.e., social vs. economic leadership).

Future scholarship is also encouraged to (a) replicate the second-order model presented herein, and (b) assess the extent to which other similar constructs fit this model. Such endeavors would grant our second-order model additional credibility, explicate the LMX construct further, and also point to whether empirical redundancy in the leadership literature extends beyond LMX measures. For example, in addition to examining and synthesizing different LMX measures, such investigations could focus on other similar leadership constructs (e.g., transformational leadership; Graen and Uhl-Bien, 1995). Such scholarship would broaden our understanding of the LMX construct by further specifying the different facets that comprised the construct and begin to tackle the general problem of construct proliferation, which is common in the behavioral sciences and not necessarily specific to the LMX arena (e.g., Harter and Schmidt, 2008; Manata and Spottswood, 2022). As suggested previously, addressing the problem of construct proliferation is decidedly important because treating interchangeable constructs as unique is likely to impede scientific progress (Joseph et al., 2011). That is, construct proliferation can create the illusion of disarray when in fact there is none. Moreover, failing to account for the presence of a second-order factor means that the incorrect measurement model is being used when attempting to estimate relationships between latent constructs. Thus, although there is value in developing and synthesizing additional measures that capture different characteristics of the LMX construct, it would be problematic to treat such factors as unique when in fact they were not.

### Limitations

First, it is important to note that this study only considered certain forms of validity, primarily structural or factorial validity. As is noted elsewhere, there are other forms of validity that are also of interest to scholars (e.g., face validity, Mosier, 1947), which some would argue are more theoretical and thus less data driven. There are, however, good reasons to believe that this limitation is not as serious as some believe it to be. In particular, it is important to recognize that elements of content validity can be summarized by tests of internal consistency and parallelism, which are the primary ways by which structural or factorial validity is assessed. That is, one reason for why items fail to fit the data is because item content is misaligned in some manner (Hunter, 1980; Boster, 2012). The clearest example of this can be seen in our second-order factor analyses, where the ELMX failed continuously to fit the data. That is, a comparison of ELMX’s content to the other eight LMX constructs shows a clear demarcation in content between social and transactional or economic elements of leadership. That is, ELMX was dropped continuously from the second-order factor cluster because subjects were interpreting the item content differently. Nevertheless, we acknowledge that establishing evidence for other kinds of validity is useful, and we encourage future scholars to continue exploring the validity of these measures with the use of different methods, considerations, and organizational samples (e.g., student workforce, Manata, 2020).

Second, an additional criticism is that items were dropped when performing the CFAs. In specific, one common belief is that dropping items when performing CFA yields an exploratory analysis, i.e., dropping items produces a new, unknown measure that is unrelated to the initial construct of interest. For example, if two scholars use LMX-7 in their respective investigations but then drop different items to attain adequate model fit, proponents of this view would argue that two different constructs were measured because different item sets were used. This would also mean that these different investigations were not directly comparable. Although this criticism may have some merit in some contexts, we do not believe that this is a major problem in the LMX arena. Martin et al.’s (2016) meta-analysis, for example, provides some support for this claim. That is, despite assessing the effects of purportedly different measures of LMX (e.g., LMX-7 and LMX-MDM), where different items are dropped regularly across different investigations, results remained homogeneous between indices. If dropping items created different measures of alternate constructs, as is suggested commonly, then homogeneity in effect sizes between measures would be unlikely. It is also worth reiterating that LMX measures were, on average, correlated very strongly. Nevertheless, we recognize that there are those that believe that dropping items from a CFA constitutes a serious limitation, and we also note that scholars’ theoretical reasoning can be flawed. As such, we reiterate the importance of replicating the results reported herein. Although, we emphasize that such replications should use the full measurement batteries, as opposed to using only those items that were retained herein. This is because items that are otherwise valid can drop from the analysis due to sampling error or specific factor variance (see Hunter, 1980).

Third, the use of alternate samples when conducting such investigations would be beneficial in that it would begin to address the limitation that stems from the similarity of our samples across all three investigations (i.e., online Qualtrics panels). Although online samples represent an advantage in that they are more diverse than traditional organizational samples (Landers and Behrend, 2015), we are not able to generalize our results to other types of samples. Such criticisms are tempered by the fact that similar conclusions have been drawn by others that have conducted independent investigations that account for different types of samples and measures (e.g., Joseph et al., 2011; Martin et al., 2016), but we concede that additional measurement work concerning these matters will help strengthen the credibility of the findings reported herein.

Finally, future research will benefit from exploring the extent to which the second-order unidimensional model remains valid across time (Boster, 2012) and between groups and levels of analysis (Dyer et al., 2005). Ultimately, the multilevel and dynamic nature of LMX indicates that future researchers will likely be concerned with the extent to which their measures of LMX remain invariant between levels of analysis and across time.

## Data Availability Statement

The raw data supporting the conclusions of this article will be made available by the authors, without undue reservation.

## Ethics Statement

The studies involving human participants were reviewed and approved by Institutional Review Board at Portland State University. Written informed consent for participation was not required for this study in accordance with the national legislation and the institutional requirements.

## Author Contributions

BM collected the data, performed the analyses, and wrote a majority of the manuscript. SG helped with data collection and writing the manuscript. Both authors contributed to the article and approved the submitted version.

## Conflict of Interest

The authors declare that the research was conducted in the absence of any commercial or financial relationships that could be construed as a potential conflict of interest.

## Publisher’s Note

All claims expressed in this article are solely those of the authors and do not necessarily represent those of their affiliated organizations, or those of the publisher, the editors and the reviewers. Any product that may be evaluated in this article, or claim that may be made by its manufacturer, is not guaranteed or endorsed by the publisher.
