# Supplementary material for: Conceptualizing Leader–Member Exchange as a Second-Order Construct
Source: Front Psychol. 2022 Jul 7;13:953860. doi: 10.3389/fpsyg.2022.953860 (PMC9301325; doi:10.3389/fpsyg.2022.953860)
Supplement: Supplementary file 1 [file Table_1.DOCX]

**S1 Table. Complete Measures (Studies 1 and 2)**

| *LMX-7* |
| --- |
| Do you know where you stand with your leader...do you usually know how satisfied your leader is with what you do? |
| How well does your leader understand your job problems and needs? |
| How well does your leader recognize your potential? |
| Regardless of how much formal authority he/she has built into his/her position, what are the chances that your leader would use his/her power to help you solve problems in your work? |
| Again, regardless of the amount of formal authority your leader has, what are the chances that he/she would “bail you out,” at his/her expense? |
| I have enough confidence in my leader that I would defend and justify his/her decision if he/she were not present to do so? |
| How would you characterize your working relationship with your leader? |

| *LMX-MDM* |  |  |
| --- | --- | --- |
| *Affect* |  |  |
| I like my supervisor very much as a person. |  |  |
| My supervisor is the kind of person one would like to have as a friend. |  |  |
| My supervisor is a lot of fun to work with. |  |  |
| *Loyalty* |  |  |
| My supervisor defends my work actions to a superior, even without complete knowledge of the issue in question. |  |  |
| My supervisor would come to my defense if I were “attacked” by others |  |  |
| My supervisor would defend me to others in the organization if I made an honest mistake |  |  |
| *Contribution* |  |  |
| I do work for my supervisor that goes beyond what is specified in my job description |  |  |
| I am willing to apply extra efforts, beyond those normally required, to further the interests of my work group |  |  |
| *Professional respect* |  |  |
| I am impressed with my supervisor’s knowledge of his/her job |  |  |
| I respect my supervisor’s knowledge of and competence on the job |  |  |
| I admire my supervisor’s professional skills |  |  |

| *LMSX* |
| --- |
| My manager and I have a two-way exchange relationship |
| I do not have to specify the exact conditions to know my manager will return a favor |
| If I do something for my manager, he or she will eventually repay me |
| I have a balance of inputs and outputs with my manager |
| My efforts are reciprocated by my manager |
| My relationship with my manager is composed of comparable exchanges of giving and taking |
| When I give effort at work, my manager will return it |
| Voluntary actions on my part will be returned in some way by my manager |
| *ELMX* |
| The most accurate way to describe my relationship with my manager is that I do what I am told to do |
| My relationship with my manager is impersonal–we don't have a personal relationship |
| I only want put in extra effort for my manager when I know in advance how he or she will repay me |
| I do what my manager demands from me, mainly because he or she is my formal boss |
| I do not care what my manager does for me in the long run, only what he or she does right now |
| I watch very carefully what I get from my manager, relative to what I contribute |
| My relationship with my manager is mainly based on authority, he or she has the right to make decisions on my behalf and I do what I am told to do |
| All I really expect from my manager is that he or she fulfils his or hers formal role as supervisor or boss |

| *SLMX* |  |  |
| --- | --- | --- |
| I don't mind working hard today–I know I will eventually be rewarded by my manager |  |  |
| I worry that all my efforts on behalf of my manager will never be rewarded (reverse scored) |  |  |
| My relationship with my manager is about mutual sacrifice, sometimes I give more than I receive and sometimes I receive more than I give |  |  |
| Even though I may not always receive the recognition from my manager I deserve, I know that he or she will take good care of me in the future |  |  |
| My relationship with my manager is based on mutual trust |  |  |
| My manager has made a significant investment in me |  |  |
| I try to look out for the best interest of my manager because I can rely on my manager to take care of me |  |  |
| The things I do on the job today will benefit my standing with my manager in the long run |  |  |
| *LMCQ* |  |  |
| With regard to getting things done, the conversations between my supervisor and me are efficient |  |  |
| When discussing work-related matters, my supervisor and I can convey a lot to each other even in a short conversation |  |  |
| When talking about work tasks, the conversations between my supervisor and me are often smooth |  |  |
| When talking about how to get things done, the conversations between my supervisor and me usually flow nicely |  |  |
| When talking about how to get things done at work, my supervisor and I usually align our ideas pretty easily |  |  |
| When talking about how to get things done at work, my supervisor and I are usually in sync with each other |  |  |
| My supervisor and I usually have accurate understanding of what the other is saying when trying to get things done at work |  |  |
| When we discuss how to get things done at work, my supervisor and I usually have no problem correctly understanding each other’s ideas |  |  |
| My supervisor and I interpret each other’s ideas accurately when discussing work-related matters |  |  |

**Complete Measures (Study 3)**

| *LMX-7* |
| --- |
| Do you know where you stand with your subordinate...do you usually know how satisfied your subordinate is with what you do? |
| How well does your subordinate understand your job problems and needs? |
| How well does your subordinate recognize your potential? |
| Regardless of how much formal authority he/she has built into his/her position, what are the chances that your subordinate would use his/her power to help you solve problems in your work? |
| Again, regardless of the amount of formal authority your subordinate has, what are the chances that he/she would “bail you out,” at his/her expense? |
| I have enough confidence in my subordinate that I would defend and justify his/her decision if he/she were not present to do so? |
| How would you characterize your working relationship with your subordinate? |

| *LMX-MDM* |
| --- |
| *Affect* |
| I like my subordinate very much as a person. |
| My subordinate is the kind of person one would like to have as a friend. |
| My subordinate is a lot of fun to work with. |
| *Loyalty* |
| My subordinate defends my decisions, even without complete knowledge of the issue in question. |
| My subordinate would come to my defense if I were “attacked” by others |
| My subordinate would defend me to others in the organization if I made an honest mistake |
| *Contribution* |
| I do work for my subordinate that goes beyond what is specified in my job description |
| I am willing to apply extra efforts, beyond those normally required, to further the interests of my work group |
| *Professional respect* |
| I am impressed with my subordinate’s knowledge of his/her job |
| I respect my subordinate’s knowledge of and competence on the job |
| I admire my subordinate’s professional skills |

| *LMSX* |  |  |
| --- | --- | --- |
| My subordinate and I have a two-way exchange relationship |  |  |
| I do not have to specify the exact conditions to know my subordinate will return a favor |  |  |
| If I do something for my subordinate, he or she will eventually repay me |  |  |
| I have a balance of inputs and outputs with my subordinate |  |  |
| My efforts are reciprocated by my subordinate |  |  |
| My relationship with my subordinate is composed of comparable exchanges of giving and taking |  |  |
| When I give effort at work, my subordinate will return it |  |  |
| Voluntary actions on my part will be returned in some way by my subordinate |  |  |
| *ELMX* |  |  |
| The most accurate way to describe my relationship with my subordinate is that he/she does what I tell him/her to do |  |  |
| My relationship with my subordinate is impersonal–we don't have a personal relationship |  |  |
| I only want to put in extra effort for my subordinate when I know in advance how he/she will repay me |  |  |
| I support my subordinate, mainly because that is my job |  |  |
| I do not care what my subordinate does for me in the long run, only what he/she does right now |  |  |
| I watch very carefully what I get from my subordinate, relative to what I contribute |  |  |
| My relationship with my subordinate is mainly based on authority, I have the right to make decisions on his/her behalf |  |  |
| All I really expect from my subordinate is that he/she fulfils his/her formal role |  |  |

| *SLMX* |  |  |
| --- | --- | --- |
| I don't mind working hard today–I know I will eventually be rewarded by my subordinate |  |  |
| I worry that all my efforts on behalf of my subordinate will never be rewarded (reverse scored) |  |  |
| My relationship with my subordinate is about mutual sacrifice, sometimes I give more than I receive and sometimes I receive more than I give |  |  |
| Even though I may not always receive the recognition from my subordinate I deserve, I know that he or she will take good care of me in the future |  |  |
| My relationship with my subordinate is based on mutual trust |  |  |
| My subordinate has made a significant investment in me |  |  |
| I try to look out for the best interest of my subordinate because I can rely on my subordinate to take care of me |  |  |
| The things I do on the job today will benefit my standing with my subordinate in the long run |  |  |
| *LMCQ* |  |  |
| With regard to getting things done, the conversations between my subordinate and me are efficient |  |  |
| When discussing work-related matters, my subordinate and I can convey a lot to each other even in a short conversation |  |  |
| When talking about work tasks, the conversations between my subordinate and me are often smooth |  |  |
| When talking about how to get things done, the conversations between my subordinate and me usually flow nicely |  |  |
| When talking about how to get things done at work, my subordinate and I usually align our ideas pretty easily |  |  |
| When talking about how to get things done at work, my subordinate and I are usually in sync with each other |  |  |
| My subordinate and I usually have an accurate understanding of what the other is saying when trying to get things done at work |  |  |
| When we discuss how to get things done at work, my subordinate and I usually have no problem correctly understanding each other’s ideas |  |  |
| My subordinate and I interpret each other’s ideas accurately when discussing work-related matters |  |  |
